# Transcriptome Analysis of Sexually Dimorphic Chinese White Wax Scale Insects Reveals Key Differences in Developmental Programs and Transcription Factor Expression

**DOI:** 10.1038/srep08141

**Published:** 2015-01-30

**Authors:** Pu Yang, Xiao-Ming Chen, Wei-Wei Liu, Ying Feng, Tao Sun

**Affiliations:** 1 Research Institute of Resources Insects, Chinese Academy of Forestry, Key Laboratory of Cultivating and Utilization of Resources Insects of State Forestry Administration, Kunming, 650224, China

## Abstract

The Chinese white wax scale insect, *Ericerus pela*, represents one of the most dramatic examples of sexual dimorphism in any insect species. In this study, we showed that although *E. pela* males display complete metamorphosis similar to holometabolous insects, the species forms the sister group to *Acyrthosiphon pisum* and cluster with hemimetabolous insects. The gene expression profile and Gene Ontology (GO) analyses revealed that the two sexes engaged in distinct developmental programs. In particular, female development appeared to prioritize the expression of genes related to cellular, metabolic, and developmental processes and to anatomical structure formation in nymphs. By contrast, male nymphal development is characterized by the significant down-regulation of genes involved in chitin, the respiratory system, and neurons. The wing and appendage morphogenesis, anatomical and tissue structure morphogenesis programs activated after male nymphal development. Transcription factors (that convey juvenile hormone or ecdysone signals, and Hox genes) and DNA methyltransferase were also differentially expressed between females and males. These results may indicate the roles that these differentially expressed genes play in regulating sexual dimorphism through orchestrating complex genetic programs. This differential expression was particularly prominent for processes linked to female development and wing development in males.

Most animal species are sexually dimorphic and display phenotypic differences in morphology, physiology, and/or behavior between females and males of the same species. Exaggerated dimorphic traits are predominantly used in the competition for mates^1^. Sexual dimorphism is believed to have arisen from differential mating processes or sexual selection^1^,^2^,^3^, but the evolutionary significance of sexual dimorphism is likely to be more complex than can be explained by these mechanisms^3^.

The Chinese white wax scale insect *Ericerus pela* (Chavannes) (Hemiptera: Coccidae) is a famous resource insect due to its role in economic production. It has been bred in China for more than a thousand years. The males are the best-known wax producers, and they secrete large amounts of pure white wax during the second-instar nymphal stage. The white wax has historically been used in traditional medicine, printing, and candle production, and its use has since expanded to the food, chemical, pharmaceutical, and cosmetic industries^4^.

Females and males are so morphologically distinct that they can be mistaken for members of different species (Figure 1). The females are neotenous and develop through egg, nymph (two instars), and adult stages (Figure 1, FE-FA3). The female eggs are reddish in color before hatching (Figure 1, FE). The female first-instar nymphs (Figure 1, FF) are phototactic and active, and they inhabit the upper sides of leaves. The second-instar nymphs (Figure 1, FS) inhabit tree branches, are immobile, and have hard chitin cuticles. They have only rudimentary legs, antennae, and simple eyes. Females change little during the transition from second-instar nymph to adult (Figure 1, FS-FA1); their shape remains the same, with an increase in body volume and a deepening of color (Figure 1, FA1–FA3). They remain immobile as adults^4^,^5^,^6^,^7^.

By contrast, males display more dramatic morphological transitions, reminiscent of the ones seen in holometabolous insects, which we describe here as “holometabolous-like”. After embryonic development, males undergo first and second-instar nymph, prepupal, pupal, and adult stages (Figure 1, ME-MA). The male eggs are light yellow in color before hatching (Figure 1, ME). The first-instar nymphs (Figure 1, MF) are lucifugous and inactive and inhabit the lower surfaces of leaves. During this stage, males are morphologically very similar to female first-instar nymphs. However, females and males inhabit different sides of the leaves because their stylet bundles are significantly different. Male second-instar nymphs (Figure 1, MS) produce a wax layer (Figure 1), a result of continued wax synthesis and secretion during the early and later second-instar nymphal stages. They are also immobile and characterized by a transparent cuticle and fat body. The male nymphs and pupae (Figure 1, MPP and MP) remain stationary until eclosion, and the pupae do not feed when they finally emerge as winged adults (Figure 1, MA)^4^,^5^,^6^,^7^. Holometabolous-like development in male *E. pela* is associated with substantial developmental changes and results in a distinctive adult form that is capable of flight and can engage in courtship behavior. *E. pela* males and females are distinctly different at each postembryonic developmental stage, and the sexually dimorphic traits accumulate gradually during the life cycle. Detailed molecular studies of these sexually dimorphic features are still lacking.

The female neotenous characteristics and male holometabolous-like development raise the possibility that the juvenile hormone (JH) and ecdysone signaling pathways may play important roles in regulating sexually dimorphic processes in scale insects. Hormone-controlled regulatory networks interact with Hox genes and cell signaling proteins to activate tissue-specific and stage-specific responses^8^,^9^,^10^,^11^,^12^. However, the regulatory networks and key signaling circuits that mediate physiognomic differences among developmental stages are still largely unknown, and no significant work has been conducted in scale insects. For instance, large-scale studies of transcriptomes or proteomes examining sexual dimorphism in coccids are lacking. Such global transcriptomic analyses are needed to begin explaining how gene expression networks are responsible for sexual dimorphism and are required for identifying genes that drive the development of sexually dimorphic forms.

In this study, we constructed ten libraries of *E. pela* at different developmental stages using RNA-Seq to provide a global transcriptomic characterization of its dimorphic sexes. By comparing gene expression profiles between females and males, we defined gene sets that build the molecular bases of sexually dimorphic development. These results will improve our understanding of sexual dimorphism in scale insects and other insect species.

## Results

### Sequence assembly and annotation of predicted proteins

One transcriptome of *E. pela* was obtained by pooling individual eggs, first/second instar nymphs, male pupae, and male and female adults. Illumina sequencing produced a total of 88,153,000 reads covering a total of 7,933,770,000 bp after removal of low-quality sequences (SRA accession number SRA047325.1). These reads were assembled into 1,481,387 contigs ranging from 50 bp to 4,489 bp (average size 112 bp; contig N50 was 90 bp). These contigs were further assembled into 231,175 scaffolds (average size 245 bp; contig N50 was 265 bp) using paired end-joining and gap-filling (Table 1). We finally obtained 156,859 unigenes with a mean length of 302 bp (contig N50 was 307 bp). The lengths of the 2,316 unigenes were above 1000 bp. A total of 32,029 unigenes (20.34% of all unigenes) provided above-cutoff (E value < 10^−5^) BLAST hits against the non-redundant (nr) database.

There were 13,257, 21,361, and 9,315 unigenes suitable for annotation in the Gene Ontology (GO), KEGG orthology (KO), and Cluster of Orthologous Groups (COG) databases, respectively, based on sequence homology (Table 1).

### Genomic divergence between hemimetabolous and holometabolous insects

**Figure 2:**
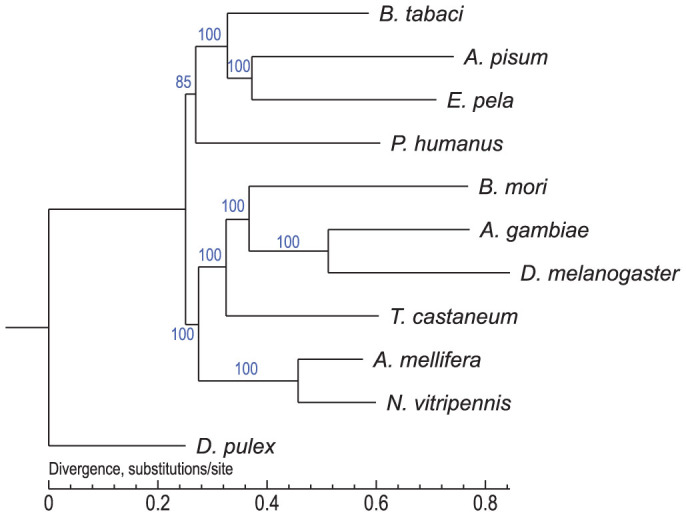
PhyML phylogenetic trees of four hemimetabolous insects and six holometabolous insects based on transcriptome data. The four hemimetabolous insects were *Bemisia tabaci*, *Acyrthosiphon pisum*, *Ericerus pela*, and *Pediculus humanus*. The six holometabolous insects were *Bombyx mori*, *Anopheles gambiae*, *Drosophila melanogaster*, *Tribolium castaneum*, *Apis mellifera*, and *Nasonia vitripennis*. The out-group was *Daphnia pulex*.

The PhyML (Phylogenetic estimation using Maximum Likelihood) phylogenetic tree (Figure 2) showed that *E. pela*, despite the males displaying complete metamorphosis similar to holometabolous insects, formed the sister group to *Acyrthosiphon pisum* and clustered with hemimetabolous insects. As demonstrated in previous studies^13^,^14^,^15^, all hemimetabolous insects clustered together with *Pediculus humanus* (the most basal of Hemimetabola) and formed a sister group to Holometabola.

### Sequencing of RNA-Seq libraries

We constructed ten RNA-Seq libraries from males and females at different developmental stages (GEO accession number GSE52228 and GSE63934), including female first- (FF) and second-instar nymphs (FS) and early- (FA1), middle- (FA2), and late-stage adults (FA3) as well as male first- (MF) and second-instar nymphs (MS), pre-pupae (MPP), pupae (MP), and adults (MA). Each of the ten RNA-Seq libraries generated over 7.0 million clean reads (Table S1).

### Differential post-embryonic developmental programs in females and males

#### Female development

**Figure 3:**
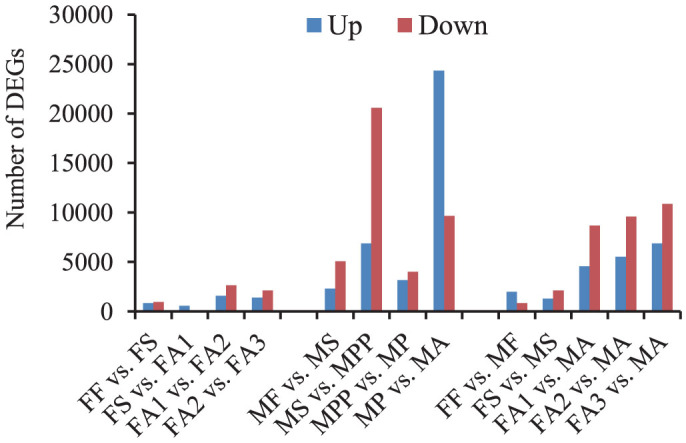
Number of DEGs in each pairwise comparison. Blue bar is up-regulated, red bar is down-regulated.

We identified 1,771 differentially-expressed genes (DEGs) between the first (FF) and second instar (FS), with 829 up-regulated and 942 down-regulated genes in FS (Figure 3). The biological process terms included muscle, cellular process, metabolic process, and anatomical structure morphogenesis (Table S2). There were only 686 DEGs (581 up-regulated, 105 down-regulated) during the second-instar molt (FS vs. FA1). The number of DEGs increased with female development (FA1vs. FA2, FA2 vs. FA3). GO analysis showed that adults down-regulated most chitin-related genes but exhibited higher activities in fat accumulation-related pathways later in development (Table S2), resulting in dramatic increases in body volume without changes in body shape. Generally, female development prioritized cellular processes, metabolic processes, and structure formation, followed by fat accumulation in late adulthood.

#### Male development

The number of DEGs during the molt from first instar (MF) to second instar (MS) (2,304 up-regulated, 5,084 down-regulated) was very different from the values in females (FF vs. FS). Male nymphal development was characterized by significant down-regulation of most GO biological process terms related to chitin, the respiratory system, and neurons and up-regulation of muscle-related terms (Table S2). There were 32 DEGs enriched in the GO molecular function term of “fatty acid activity”, which were not associated with female development, and more DEGs were enriched in the fat acquisition pathways than in females (Table S2–3). The number of DEGs increased dramatically during the molt from second-instar (MS) to prepupae (MPP) and the transition from pupae (MP) to adult (MA) (Figure 3). Many DEGs enriched in the GO term of wing morphogenesis, appendage morphogenesis, and cytoskeleton. Anatomical and tissue structure development terms were identified during the molt from prepupae (MPP) to pupae (MP). These results support the two different developmental programs observed in females and males.

### Transcriptional changes among developmental stages

There were a total of 2,817 DEGs between the FF and MF libraries, with 1,984 up-regulated and 833 down-regulated genes in MF (Figure 3). There were 10 DEGs enriched in the biological process term related to “compound eye photoreceptor development”, all up-regulated in males, which was consistent with the lucifugous physiological characterization of male nymphs. The number of DEGs increased to 3,410 (1,294 up-regulated and 2,116 down-regulated genes) when FS was compared to MS. Most biological process terms were related to chitin and were largely down-regulated in males; this result is probably related to the transparent cuticle of male second-instar nymphs. Many DEGs were enriched in “metabolic pathways” (314), “lipid metabolism” (208), “insulin signaling pathway” (49), and “steroid hormone biosynthesis” (37) (Table S2–3). These results suggest that pathways involved in fat metabolism and storage were used differently between female and male nymphs.

When female adults at different stages were compared to male adults (MA), the number of DEGs increased in each comparison. Most DEGs were enriched in molecular function terms related to “catalytic activity” and “hydrolase activity”. Additionally, many DEGs were enriched in “somatic muscle development”, “fatty acid synthase activity”, “DNA or RNA related process”, and many other GO terms. The KEGG pathway annotation yielded similar results, but “insulin signaling pathway” and “steroid hormone biosynthesis” were also found (Table S2–3).

### qRT-PCR validation

The gene expression profiles were validated by qRT-PCR. The results were closely consistent with the RNA-Seq results (Combination Chi-square = 115.6168, *df* = 20, P < 0.01). The discrepancies at a few developmental stages between the qRT-PCR and RNA-Seq results may have been caused by a sensitivity bias between the two methods or by the use of different statistical methods and threshold values in qRT-PCR and RNA-Seq.

### Gene expression analysis

**Figure 4:**
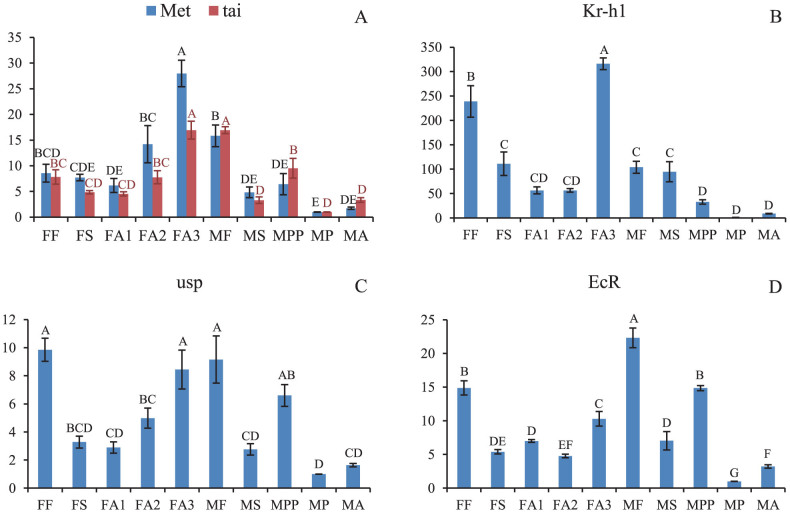
Relative quantification of the gene expression levels of five transcription factors (that convey JH and ecdysone signals) across different developmental stages. Samples marked with the same letter were not significantly different from each other, *P* > 0.01. A: Met and tai; B: Kr-h1; C: usp; D: EcR. FF: female first-instar nymphs; FS: female second-instar nymphs; FA1: early-stage female adults; FA2: middle-stage female adults; FA3: late-stage female adults; MF: male first-instar nymphs; MS: male second-instar nymphs; MPP: male pre-pupal stage; MP: male pupal stage; MA: male adults.

Methoprene-tolerant protein (Met) conveys the JH signal to prevent precocious metamorphosis through the maintenance of high expression of Kruppel homolog 1 (Kr-h1)^16^,^17^,^18^. taiman (tai) is currently the best-known protein partner of Met^19^. We found that Met and tai expression levels did not change significantly during the molt from first -instar (FF) to second -instar (FS) and the transition from second-instar (FS) to adult (FA1) but increased significantly in developed female adults (FA3). Met and tai decreased substantially after the first instar in males (Figure 4A). The expression pattern of Kr-h1 differed between females and males, with opposing expression patterns in females and males during the molt from first -instar to second -instar and the transition from second-instar to adults (FA1) or prepupae (MPP) (Figure 4B).

Functional molting hormone receptors consist of the products of two genes, ecdysone receptor (EcR) and ultraspiracle (usp)^20^, with usp activation dependent on EcR^21^. usp and EcR showed different expression profiles in females and males. usp and EcR did not change significantly during female metamorphosis (Figure 4C, D).

**Figure 5:**
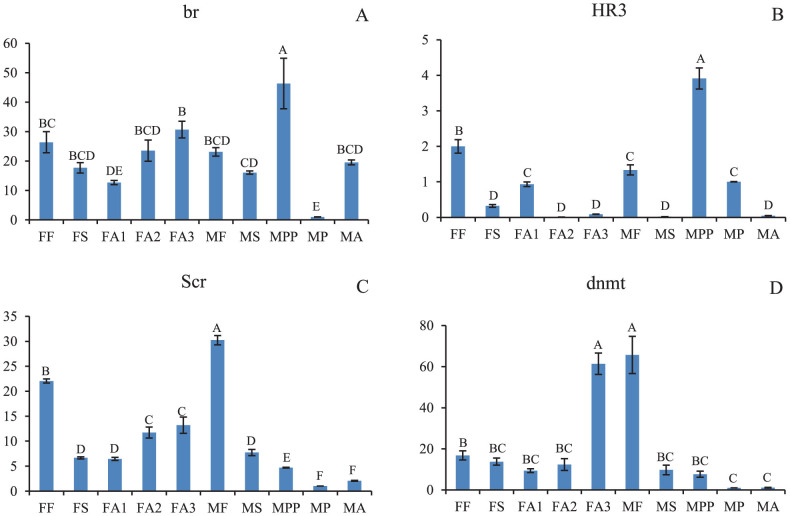
Relative quantification of four gene expression levels across different developmental stages. Samples marked with the same letter were not significantly different from each other, *P* > 0.01. A: br; B: HR3; C: Scr; D: dnmt. FF: female first-instar nymphs; FS: female second-instar nymphs; FA1: early-stage female adults; FA2: middle-stage female adults; FA3: late-stage female adults; MF: male first-instar nymphs; MS: male second-instar nymphs; MPP: male pre-pupal stage; MP: male pupal stage; MA: male adults.

The expression of broad (br), which regulates wing development, was enhanced by JH in *Blattella germanica*^17^. In this study, we detected br expression during each developmental stage of *E. pela*. It exhibited the highest expression level during the male prepupal stage (MPP) (Figure 5A). Hormone receptor 3 (HR3) has been shown to be responsible for the coordination of the parts of the transcriptional program that control metamorphosis^22^,^23^,^24^ and increased markedly during the male prepupal stage in *E. pela*, consistent with its role in the regulation of the metamorphosis cascade (Figure 5B).

Hox genes, which are related to limb development (Antp, Antennapedia), wing development (Scr, Sex combs reduced), and segmentation formation (abd-A, abdominal A; Abd-B, Abdominal B; en, engrailed; Ubx, Ultrabithorax), are key factors in establishing differential structures^9^,^10^,^11^,^12^. The qRT-PCR analysis of Scr expression revealed striking differences between females and males (Figure 5C). However, our present results cannot fully explain the functions of other Hox genes in *E. pela*.

One mechanism that contributes to sex determination in scale insects is paternal chromosome heterochromatization^25^. The DNA methyltransferase (dnmt) gene was found to play a key role in DNA methylation and was considered a molecular counterpart of imprinting in mealybugs^26^. Thus, we assessed the expression dynamics of the dnmt gene in *E. pela* and found its expression level increased sharply at the developed adult stage (FA3), in which the embryos have begun development. It also exhibited pronounced expression in male first-instar nymphs (MF) that decreased progressively with development (Figure 5D).

## Discussion

*E. pela* females and males have different physiological traits and ecological strategies. The formation of the cuticle, metamorphosis, and physiological differences play pivotal roles in their sexually dimorphic development.

The morphological differences between male and female *E. pela* are visible in the cuticle. The appearance and properties of the cuticles depend mostly on chitin and cuticle proteins^27^,^28^,^29^,^30^. During the first-instar nymphal stage, males are morphologically very similar to females. However, during their second-instar nymphal stage, females develop hard chitin cuticles, while males are coated with thick wax layers. We found down-regulation of all DEGs linked to the GO terms “chitin biosynthetic process” and “chitin metabolic process” in male second-instar nymphs compared to male first-instar nymphs. This gene regulation results in the soft, transparent cuticles that need the protection of the wax layer. Unlike male second-instar nymphs, female second-instar nymphs up-regulate most of the DEGs enriched in the GO terms “chitin biosynthetic process”, “chitin metabolic process”, and “polysaccharide metabolic process” (Table S2), which are closely related to the hard chitin cuticle coats of females. These DEGs resulted in the distinctly different cuticles between females and males.

The fat body of insects is a key organ in metabolism, including energy storage and synthesis of carbohydrates, lipids, and proteins^31^. In this study, the GO terms “fatty acid synthase activity” and “catalytic activity” were present in all pairwise comparisons (Table S2). The KEGG pathways related to “fatty acid biosynthesis”, “steroid hormone biosynthesis”, and “peroxisome” were similarly present in all comparisons. Similar results were also found for *Bemisia tabaci*^32^, where these terms were closely related to fat accumulation. During fat body and ovary development, the shape of the female adults did not change, although the body volume increased and the color deepened^5^. This fact is closely related to the characterization of fat synthesis during female adult development.

The nature of insect molting depends on JH^18^. Met and Kr-h1 repress precocious metamorphosis of larvae in hemimetabolous and holometabolous insects^16^,^17^,^18^,^19^. Met and tai are required for adult female reproduction in *Pyrrhocoris apterus*^19^. In this study, we found differences in the Met and Kr-h1 expression profile between female and males, which may drive sexually dimorphic development. The high expression levels of Met and tai in developed female adults (FA3) may imply their requirement for reproduction. However, their expression profiles cannot fully explain their functions in *E. pela*.

Male adult wing development is a major innovation compared with females in *E. pela*. The br genes regulate wing development in hemimetabolous insects, such as *Blattella germanica* and *Oncopeltus fasciatus*^17^. In this study, we found that the expression levels of most br genes did not change during the female second-instar nymphal stage. By contrast, certain br genes exhibited increased expression levels during the male second-instar nymphal stage (Table S4, Figure 5A). The role of the Scr gene in wing repression is conserved in *O. fasciatus* and other insects. Scr-depletion restarted the wing program in *O. fasciatus*^33^,^34^. In our work presented here, we found that Scr was highly expressed during the male first-instar nymphal stage and that its expression decreased markedly in the male prepupal, pupal and adult stages (Figure 5C) in which wings are formed. We also noticed that the vestigial (vg) gene exhibited significantly higher expression in male prepupae, pupae, and adults compared with other developmental stages (Table S4). The vg gene has been reported to be a key regulator of wing development and shown to be induced by the activation of *Notch* signaling^35^. We believe that the br genes, Scr gene and vg gene are important for sexual dimorphism in wing development of *E. pela*.

Sexual dimorphism is closely related to sex determination. It orchestrates complex genetic programs that control the sexually dimorphic traits subsequently expressed throughout the life cycle. In scale insects, sex determination is based on genomic imprinting of an entire set of paternally contributed chromosomes^25^,^36^. According to Bongiorni et al. (2007), heterochromatization during embryogenesis in male *Planococcus citri* is due to maternal determination^37^, whereas Buglia & Ferraro (2004) suggested a paternal sex-determination mechanism^38^. According our qRT-PCR results, the highest level of dnmt expression was in male first-instar nymphs (MF) and developed females (FA3) (Figure 5D) when the embryos had begun to develop. DNA methylation is closely related to sex ratios in scale insects. However, it is difficult to explain how the female and male *E. pela* offspring are produced at a nearly 1:2 ratio. Furthermore, the sex ratios vary in different geographical populations. The mechanism by which DNA methylation is regulated will likely elucidate the mechanism underlying sex differentiation in scale insects.

## Methods

### Insect handling and RNA isolation

*E. pela* were reared in Chinese privet (*Ligustrum lucidum*) trees in the Research Institute of Resources Insects. *E. pela* from each developmental stage were collected for the pooled transcriptome. Thousands of eggs without distinguishing between females and males were collected for the pooled transcriptome. For the first-instar nymphal stage, more than 500 female and 500 male individuals were collected. During this stage, females and males inhabited different sides of the leaves, making it is easy to distinguish females from males. More than 100 female and 100 male second-instar nymphs were collected. For the developed female adults (FA3), approximately 20 individuals were collected. For the other developmental stages, approximately 50–100 individuals of each sex were collected. The pooling approach was used for sample collection^39^,^40^. Equivalent total RNA from each stage was pooled for transcriptome sequencing. The samples of each developmental stage were washed three times in DEPC-treated (diethypyrocarbonate) water and homogenized in TRIZOL (Invitrogen, U.S.) separately. The total RNA from each sample was extracted separately according to the manufacturer's protocol. The integrity of isolated RNA was confirmed using the Agilent 2100 Bioanalyzer (Agilent Technologies, California, U.S.) with RNA integrity number (RIN) and clear characteristic peaks at 28S and 18S.

### Library Preparation for Illumina sequencing

A pooled RNA sample (20 μg) of every developmental stage (female and male) was used for cDNA library preparation. The library was constructed using the same techniques as in our previous study^4^. The library was sequenced using Illumina HiSeq™ 2000. The raw data were deposited in SRA (the sequence read archive) of the National Center for Biotechnology Information (NCBI).

### Comparative genomic analysis

The CDS and protein sequences of six holometabolous insect species (*Drosophila melanogaster* (Diptera), *Anopheles gambiae* (Diptera), *Bombyx mori* (Lepidoptera), *Tribolium castaneum* (Coleoptera), *Apis mellifera* (Hymenoptera), and *Nasonia vitripennis* (Hymenoptera)) were downloaded from the following websites, respectively: ftp.flybase.net(dmel_r5.27); http://www.vectorbase.org/Anopheles_gambiae(AgamP3.6); http://www.silkdb.org/silkdb/(release-2.0); ftp://bioinformatics.ksu.edu/pub/BeetleBase(version3.0); http://hymenopteragenome.org/drupal/sites/hymenopteragenome.org.beebase/(release-2.0); and http://genomes.arc.georgetown.edu/nasonia/nasonia_genome_consortium (OGSv1.2). Sequences from three other hemimetabolous insects (*Pediculus humanus* (Phthiraptera), *Acyrthosiphon pisum* (Hemiptera), and *Bemisia tabaci* (Homoptera)) were downloaded from the following websites, respectively: https://ftp.vectorbase.org/public_data/organism_data/phumanus); http://www.aphidbase.com/ (version 1); and http://www.ncbi.nlm.nih.gov/bioproject/PRJNA50499. Sequences from *Daphnia pulex* were downloaded from http://wfleabase.org. Alternative splicing was filtered out of the CDS, and the longest sequence was reserved.

The TreeFam method^41^,^42^ was used to classify the protein coding genes from 11 insects into narrowly-defined gene lineages. The first step was assignment of pairwise relationships. Briefly, an all-to-all protein sequence alignment was performed using Blastp (version2.2.23) with an e-value < 1e-10. The fragment alignments for each gene pair were conjoined using SOLAR (version 0.9). Two genes were termed connected if the aligned region size ratio for both genes was > 1/3. The similarity between two genes was weighted by an Hscore that ranged from 0 to 100. The second step was extracting gene families. Genes were clustered by Hcluster_sg (version1.9.2) with the following parameters: Hscore > 10, minimum edge density (total number of edges/theoretical number of edges) > 1/3. The process of clustering a gene family stopped if the family already included one or more genes from the out-group.

The phylogenetic tree was reconstructed based on the identified 382 single-copy gene families that were present in all species. The corresponding amino acid sequences were aligned with the MUSCLE (v3.8.31) program and then trimmed to remove ambiguous sites or missing data. The CDS results were aligned using the protein alignment result as a guide. The CDS alignments for each gene were then concatenated into a super-alignment containing 599,412 positions^43^. Codon sequences were extracted from the CDS alignments and used as input for building trees. Next, PhyML (http://code.google.com/p/phyml/) was applied to these sequence sets to build phylogenetic trees under the HKY85 + invgamma model for nucleotide sequences. We used 100 rapid bootstrap replicates to assess branch reliability.

### Preparation and sequencing of RNA-Seq libraries

The mRNA of each sample (FF, FS, FA1, FA2, FA3, MF, MS, and MA) was enriched separately using oligo (dT) magnetic beads after extracting the total RNA from each sample. The mRNA was fragmented (approximately 200 bp) after the addition of fragmentation buffer. First-strand cDNA was synthesized using random hexamer-primers using the mRNA fragments as templates. Buffer, dNTPs, RNase H, and DNA polymerase I were added to synthesize the second strand. The double-stranded cDNA was then purified using a QiaQuick PCR extraction kit and washed using EB buffer for end repair and the addition of single adenines. Sequencing adaptors were ligated to the fragments, and the required fragments were purified by agarose gel electrophoresis and enriched by PCR amplification. Finally, ten RNA-Seq libraries were constructed and sequenced using Illumina HiSeq™ 2000.

### Alignment with references and assessment of sequencing quality

The original image data were transformed into sequence data by base calling, which is defined as raw reads. To obtain clean reads, reads with adaptors, reads with more than 10% unknown bases, and low-quality reads were removed prior to data analysis. The clean reads were mapped to reference sequences using SOAPaligner/soap2^44^. Mismatches of no more than 2 bases were allowed in the alignment.

The sequences were assessed by read quality, statistics of alignment analysis, sequencing saturation analysis, distribution of reads on the reference gene, and distribution of reads on the reference genome. This analysis constitutes the general information of the study.

### Statistics regarding gene expression level, screening of differentially expressed genes, and enrichment analysis

Gene expression levels were calculated by the numbers of reads mapped to the reference sequence for every gene based on the RPKM (reads per kb per million reads) method^45^. To reduce the risk of false discovery and prevent the elimination of genes that play a transcriptional role, an RPKM cutoff value of 0.3 was set to delete low-abundance unigenes in the background transcriptome^46^. Information related to genes, such as coverage, symbols, and function, is also provided.

To identify differentially expressed genes (DEGs) between two samples, the clean tag frequency in each RNA-Seq library was statistically analyzed according to a strict algorithm developed from the significance of digital gene expression profiles^47^. The false discovery rate (FDR) was used to determine the threshold of *P*-values in multiple tests. We used stringent criteria (FDR ≤ 0.001 and the absolute value of log_2_ ratio ≥ 1 as the threshold) to determine significant differences in gene expression^48^.

The DEGs were chosen for further analysis of GO and KO enrichment. DEGs were first mapped to GO terms in the database to calculate the number of genes for every term. Then, the GO terms that were significantly enriched in the DEGs relative to the genomic background were found using a hypergeometric test. The formula is as equation (1).
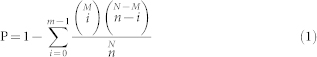
Here, N represents the total number of genes with GO annotations; n represents the number of DEGs in N; M represents the total number of genes annotated to the certain GO terms; and m represents the number of DEGs. The calculated *P*-value underwent Bonferroni Correction, and the corrected *P*-value ≤ 0.05 was chosen as a threshold. GO terms fulfilling this condition were defined as significantly enriched GO terms in DEGs^49^.

Pathway enrichment analysis provides significantly enriched signal transduction pathways, metabolic pathways, and other pathways in DEGs relative to the transcriptome background. The formula used in this analysis is the same as the one used for GO enrichment, with N representing the total number of genes with KEGG annotations (Kyoto Encyclopedia of Genes and Genomes)^50^, n representing the number of DEGs in N, M representing the total number of genes annotated to specific pathways, and m representing the number of DEGs in M^49^,^51^.

### Quantitative real-time PCR (qRT-PCR) analysis

To validate the results of RNA-Seq analysis, we performed qRT-PCR experiments with nine genes. The concentration of total RNA was determined using the Agilent 2100 Bioanalyzer (Agilent Technologies, California, U.S.). One μg of total RNA from each sample was reverse-transcribed separately in a 20 μl reaction system according to the protocol provided with the M-MLV first-strand kit (Invitrogen, China). Then, qRT-PCR was performed using SsoFast™ EvaGreen Supermix (Bio-RAD, U.S.). β-actin was used as an internal standard. Three replicates were conducted for each developmental stage. The relative expression levels of each gene were calculated using the 2^−ΔΔCt^ method^52^. The results were statistically analyzed with the DPS statistical software using the least significant difference (LSD) test at the *P* = 0.01 level^53^. The correlation between qRT-PCR and RNA-Seq data was checked based on the combination of probabilities from tests of significance^54^.

## Author Contributions

P.Y. and X.M.C. conceived and designed the experiments. P.Y. wrote the paper. P.Y. and Y.F. analyzed the data. W.W.L. and T.S. performed the qRT-PCR analysis. All authors reviewed the manuscript.

## Supplementary Material

Supplementary Information Supplementary Information

Supplementary Information Dataset 1

Supplementary Information Dataset 2

Supplementary Information Dataset 3

Supplementary Information Dataset 4

## Figures and Tables

**Figure 1:**
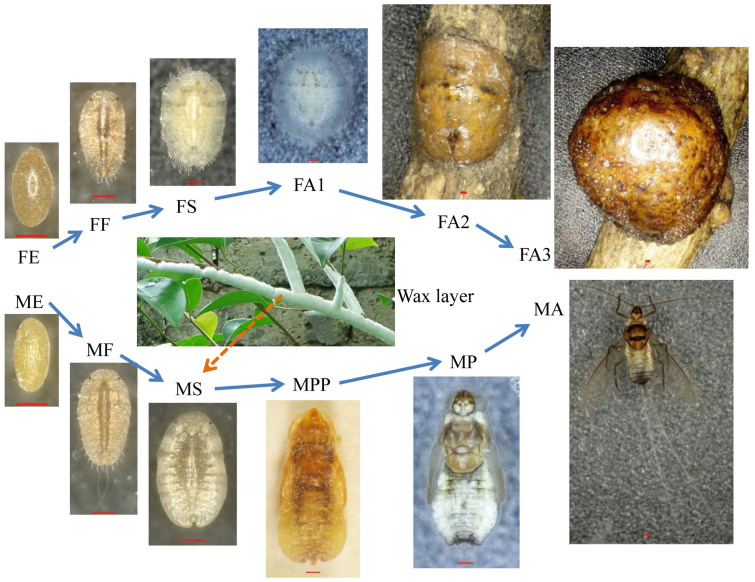
Females and males of *Ericerus pela* at different developmental stages. The females develop through an egg stage (FE), first/second instar nymph (FF, FS), early- (FA1), middle- (FA2), and later-stage adults (FA3). The males develop through an egg stage (ME), first/second instar nymphs (MF, MS), pre-pupae (MPP), pupae (MP), and adults (MA). The red bar in the figure of each stage is 0.2 mm.

**Table 1:** Summary of the *Ericerus pela* transcriptome with each developmental stage pooled

Total base pairs (bp)	7,933,770,000
Total number of reads	88,153,000
Average read length	90
Total number of contigs	1,481,387
Mean length of contigs	112
Total number of scaffolds	231,175
Mean length of scaffolds	245
Total number of unigenes	156,859
Mean length of unigenes	302
Number of unigenes with E-value < 1E-5	32,029
Number of unigenes annotated in GO	13,258
Number of unigenes annotated in KO	21,361
Number of unigenes annotated in COG	9,314
Number of unigenes annotated in Swisprot	25,261
