# Supplementary material for: Transcriptome Analysis of Sexually Dimorphic Chinese White Wax Scale Insects Reveals Key Differences in Developmental Programs and Transcription Factor Expression
Source: Sci Rep. 2015 Jan 30;5:8141. doi: 10.1038/srep08141 (PMC4311254; doi:10.1038/srep08141)
Supplement: Supplementary Information [file srep08141-s1.pdf]

Transcriptome Analysis of Sexually Dimorphic Chinese White Wax Scale Insect Reveals Key Differences in Developmental Programs and Transcription Factor Expression

Pu Yang[a], Xiao-Ming Chen[b]*, Wei-Wei Liu[c], Ying Feng[d], Tao Sun[e]

Research Institute of Resources Insects, Chinese Academy of Forestry, Key Laboratory of Cultivating and Utilization of Resources Insects of State Forestry Administration, Kunming, 650224, China

Email correspondence: [a]zjuyangpu@aliyun.com, [b]cafchxm@tom.com, [c]ypu910@126.com, [d]iugogo@126.com, [e]13033334807@163.com.

*Corresponding Authors:

Dr. Xiao-Ming Chen

Research Institute of Resources Insects

Chinese Academy of Forestry

Kunming, 650224, China

Phone: 86-871-63860019

Fax: 86-871-63860019

Email: cafchxm@tom.com

SUPPORTING INFORMATION

TABLES

Table S1. Alignment statistics for the ten RNA-Seq libraries.

Table S2. GO classification of the DEGs in each pairwise comparison.

Table S3. KO classification of the DEGs in each pairwise comparison.

Table S4. Expression levels of genes at different developmental stages of *Ericerus pela*. The expression level is indicated by the RPKM value. To validate gene expression, certain gene expression levels were also presented as copy numbers obtained by qRT-PCR.
